# Novel insights into the progression and prognosis of the calpain family members in hepatocellular carcinoma: a comprehensive integrated analysis

**DOI:** 10.3389/fmolb.2023.1162409

**Published:** 2023-07-12

**Authors:** Dongjun Dai, Dehao Wu, Runliang Ni, Ping Li, Zhifeng Tian, Yongjie Shui, Hanguang Hu, Qichun Wei

**Affiliations:** ^1^ Department of Radiation Oncology, The Second Affiliated Hospital, Zhejiang University School of Medicine, Hangzhou, China; ^2^ The Key Laboratory of Cancer Prevention and Intervention, China National Ministry of Education, Zhejiang University School of Medicine, Hangzhou, China; ^3^ Department of Oncology, The Second Affiliated Hospital, Zhejiang University School of Medicine, Hangzhou, China

**Keywords:** hepatocellular carcinoma, The Cancer Genome Atlas, B cells, immune checkpoint inhibitors, 13 immunotherapy, drug sensitivity, least absolute shrinkage and selection operator, cancer prognosis

## Abstract

**Objectives:** The goal of our bioinformatics study was to comprehensively analyze the association between the whole calpain family members and the progression and prognosis of hepatocellular carcinoma (HCC).

**Methods:** The data were collected from The Cancer Genome Atlas (TCGA). The landscape of the gene expression, copy number variation (CNV), mutation, and DNA methylation of calpain members were analyzed. Clustering analysis was performed to stratify the calpain-related groups. The least absolute shrinkage and selection operator (LASSO)-based Cox model was used to select hub survival genes.

**Results:** We found 14 out of 16 calpain members expressed differently between tumor and normal tissues of HCC. The clustering analyses revealed high- and low-risk calpain groups which had prognostic difference. We found the high-risk calpain group had higher B cell infiltration and higher expression of immune checkpoint genes HAVCR2, PDCD1, and TIGHT. The CMap analysis found that the histone deacetylase (HDAC) inhibitor trichostatin A and the PI3K-AKT-mTOR pathway inhibitors LY-294002 and wortmannin might have a therapeutic effect on the high-risk calpain group. The DEGs between calpain groups were identified. Subsequent univariate Cox analysis of each DEG and LASSO-based Cox model obtained a calpain-related prognostic signature. The risk score model of this signature showed good ability to predict the overall survival of HCC patients in TCGA datasets and external validation datasets from the Gene Expression Omnibus database and the International Cancer Genome Consortium database.

**Conclusion:** We found that calpain family members were associated with the progression, prognosis, and drug response of HCC. Our results require further studies to confirm.

## 1 Introduction

Hepatocellular carcinoma (HCC) is the sixth common malignancy worldwide (Sung et al., 2021). The overall 5-year survival rate for HCC is around 20% (Siegel et al., 2022). Barcelona Clinic Liver Cancer (BCLC) is the most commonly used staging system for HCC which considers tumor burden, Child–Pugh system, and performance status (Llovet et al., 1999). Potentially curative treatments such as surgery, chemoembolization, and radiofrequency ablation are available for limited-stage HCC patients (BCLC 0/A/B), while BCLC patients usually receive systemic therapies (Rizzo et al., 2021).

Since HCC is highly chemorefractory, several target therapy drugs were developed for treating it. Sorafenib, a small multikinase inhibitor, was first approved by the FDA (Food and Drug Administration) for treating patients with advanced stage HCC in 2007 (Cheng et al., 2009). In 2018, the REFLECT trial demonstrated that lenvatinib showed superiority over sorafenib for advanced HCC (Kudo et al., 2018). With the emergence of immune therapy, a study showed that a combination of immune checkpoint inhibitors (ICI) atezolizumab and antiangiogenic agent bevacizumab showed an improvement of 13.2% in 1 year-overall survival (OS) compared to sorafenib (Finn et al., 2020; Viscardi et al., 2022). Recently, a study showed that tremelimumab plus durvalumab (STRIDE) had a median OS of 16.43 months compared to sorafenib with a median OS of 13.77 months in unresectable HCC (Kudo, 2022). However, HCC is a highly heterogeneous cancer with substantial genomic heterogeneity, which influences the cellular response of systemic therapy such as sorafenib and ICIs (Atwa et al., 2021). For patients with resistance to sorafenib (first-line therapy) in advanced HCC, regorafenib (RESORCE), cabozantinib (CELESTIAL), ramucirumab (REACH-2), nivolumab in combination with ipilimumab (CheckMate 040), or pembrolizumab (Keynote 240) were recommended (Solimando et al., 2022). However, the improvement of OS was limited in the second-line treatment. It is important to explore the underlying genetic mechanisms of HCC that are related to prognosis and therapy response and develop new drugs targeted to advanced HCC patients.

The calpains are a family of cytoplasmic conserved cysteine proteases that catalyze the controlled proteolysis of specific substrates (Spinozzi et al., 2021). Calpains participate in a series of cancer-related processes, including cell cycle, cellular proliferation, migration, cancer cell invasion, metastasis, and apoptosis (Nian and Ma, 2021; Spinozzi et al., 2021). Several members of the calpain family were identified to be associated with cancer progression. For example, the expression of CAPN1 was positively correlated with worse prognosis of colorectal cancer and breast cancer patients (Pu et al., 2016; Xu et al., 2019). CAPN2 was found to upregulate the AKT/mTOR pathway to promote the proliferation of prostate cancer (Strom et al., 2017). CAPNS1 was observed to active the Wnt/β-catenin pathway to increase colorectal cancer progression (Cheng et al., 2018). CAPN6 was found to promote the fate of sarcoma stem cells by promoting autophagy and preventing senescence (Andrique et al., 2018). In HCC, CAPN2 was found to proteolyze PTP1B to promote β-catenin expression and activity via directly phosphorylating β-catenin and through an indirect PI3K/AKT-dependent manner to increase HCC progression (Ma et al., 2022). CAPNS1 contributes to cancer growth and metastasis of HCC by activation of the FAK-Src signaling pathway (Dai et al., 2014). However, there was no comprehensive study focusing on the association between the whole calpain family members and HCC progression and prognosis.

In our study, by utilizing The Cancer Genome Atlas (TCGA) database, we comprehensively analyzed the association between the calpain family members and progression and prognosis of HCC via integrated analysis of gene expression, copy number variation, DNA methylation, and mutation. We identified two groups of HCC patients with different expression patterns of calpain family members from the TCGA database, which had significantly different OS. Several comparisons were performed between the two groups, including the immune microenvironment, genome profile, and drug sensitivity. A calpain-related signature was identified to well-predict the OS of HCC patients in the TCGA, which was validated by external databases from the Gene Expression Omnibus (GEO) and the International Cancer Genome Consortium (ICGC).

## 2 Methods

### 2.1 The data collection

The TCGA data of RNA transcription, copy number variation and DNA methylation, and ICGC RNA expression were collected from the Xena database (Mary Goldman et al., 2019). The TCGA mutation data were obtained from the R package “TCGAmutation.” The RNA read count data were normalized by the “TMM” method (McCarthy et al., 2012) and transformed by the “voom” method by the R package “limma” (Ritchie et al., 2015). The calpain family comprised the classical calpains, which included CAPN1, CAPN2, CAPN3, CAPN8, CAPN9, CAPN11, CAPN12, CAPN13, and CAPN14; and non-classical calpains, which included CAPN5, CAPN6, CAPN7, CAPN10, and CAPN15; and small regulatory subunits CAPNS1 and CAPNS2. The GEO datasets were downloaded from the GEO database. The RNA transcription data of ICGC were obtained from the Xena database.

### 2.2 The landscape description of calpain members in OSCC

The heatmap and violin plots were used to visualize the expression difference of calpain members between tumor and normal tissues in HCC patients. The heatmap was used to visualize the association between the expression of calpain members and HCC clinical features that comprised age, gender, and TNM stage. The dumbbell plot and waterfall plot were used to represent the distribution of CNV and mutation of calpain members, respectively. The violin plot was used to exhibit the association between CNV and the gene expression of each calpain member. The KM method was applied to the survival analysis of the gene expression of each calpain member. The correlation analysis of DNA methylation and RNA expression of each calpain member was performed. For the DNA methylation analysis, we only included the probe that was targeted to the promoter region of the gene.

### 2.3 The clustering analysis

The clustering analysis was conducted by the R package “ConsensusClusterPlus” to stratify the calpain-related groups. The parameter settings were as follows: reps = 1000, pItem = 0.8, pFeature = 1, and distance = Euclidean. The survival analysis showed that the two calpain-related groups had significant prognostic difference. We, therefore, named the two groups as high- and low-risk calpain-related groups.

### 2.4 The comparisons between high- and low-risk calpain-related groups

Several comparisons were performed between the two calpain-related groups, which comprised immune microenvironment difference, differentially expressed gene (DEGs) analysis, and drug response analysis.

The immune microenvironment was assessed in several ways. The immune and stromal scores were calculated by the ESTIMATE algorithm from the R package “estimate”. The immune cell fractions were estimated by xCell, quanTIseq, EPIC, and TIMER algorithms from the R package “immunedeconv”. Furthermore, the expression of eight well-studied immune checkpoint genes, which comprised CD274, CTLA4, HAVCR2, LAG3, PDCD1, PDCD1LG2, SIGLEC15, and TIGIT, was compared between calpain-related groups.

The DEGs between calpain-related groups were analyzed by the R package “limma”. We defined the DEGs as the genes with a fold change more than 2 and a *p*-value less than 0.05. The volcano plot was used to visualize the results of DEG analysis. GSEA analysis was performed between calpain-related groups based on the log fold change from DEG analysis through the R package “clusterProfiler”. The GSEA analysis comprised items from gene ontology (GO) and Kyoto Encyclopedia of Genes and Genomes (KEGG).

The drug response difference between the calpain-related groups was analyzed by Connectivity Map (CMap) analysis (Subramanian et al., 2017) from the R package “DrInsight” (Chan et al., 2019). The calculated statistic t-test scores from DEG analysis were used as input data for CMap analysis. The drugs with a false discovery rate (FDR) < 0.1 were considered potential drugs targeted to the high-risk calpain group.

### 2.5 The prognostic model construction

Univariate Cox analysis was applied to each DEG. The DEGs significantly associated with the OS of HCC were selected for a further least absolute shrinkage and selection operator (LASSO) analysis to acquire the hub survival genes (with 10- fold cross-validation). The hub survival genes were used to build a risk score prognostic model by using the following formula: (βi × Expi) (i was the number of hub survival-related genes). The KM method was used to evaluate the prognostic value of this risk score model. The area under the receiver operating characteristic curve (AUC) plots were built by the R package “timeROC” to estimate the prediction ability of the risk score model of the 1-year, 2-year, and 3-year OS of HCC patients. This risk score model was validated by external datasets from the GEO database (GSE76427 (Grinchuk et al., 2018) and GSE54236 (Dituri et al., 2020)) and the ICGC dataset. Furthermore, a nomogram was also built to predict the 1-year, 2-year, and 3-year OS of HCC patients. The validation was performed by the calibration curve.

### 2.6 Statistical analyses

All the statistical analyses were performed by R-4.1.3. The heatmap was plotted by the R package “pheatmap”. The KM method was performed by the R package “survminer”. LASSO analysis was conducted by the R package “glmnet”. The violin plots, dumbbell plot, and KM plots were all drawn by the R package “ggplot2”. Wilcoxon analysis was performed for comparisons between two groups. A *p*-value less than 0.05 was considered statistically significant.

## 3 Results

### 3.1 The landscape of calpain members in HCC

**FIGURE 1:**
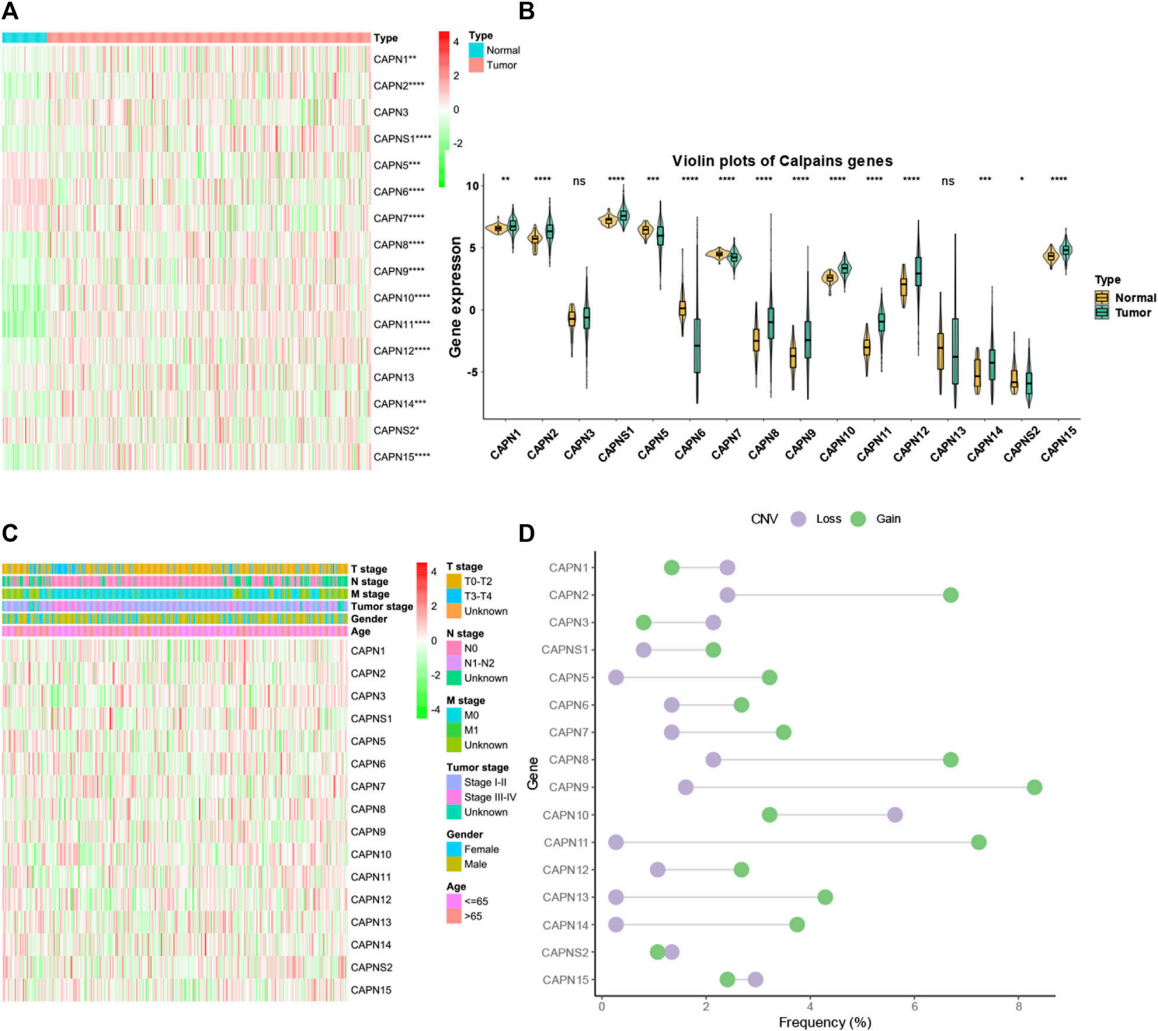
Landscape of gene expression and CNV of calpain members in HCC. **(A)** Heatmap of the gene expression level of calpain members between normal (N) and tumor (T) tissues of HCC in TCGA. **(B)** Violin plots which presented the expression of each calpain gene between normal and cancer tissues of HCC in TCGA. **(C)** Expression of calpain members in HCC with clinicopathological features that comprised age, gender, T stage, M stage, N stage, and tumor stage. **(D)** Dumbbell plot which represents the CNV distribution of calpain members in the TCGA database. The asterisks represented the statistical *p*-value (*p < 0.05; **p < 0.01; ***p < 0.001; ****p < 0.0001).

**FIGURE 2:**
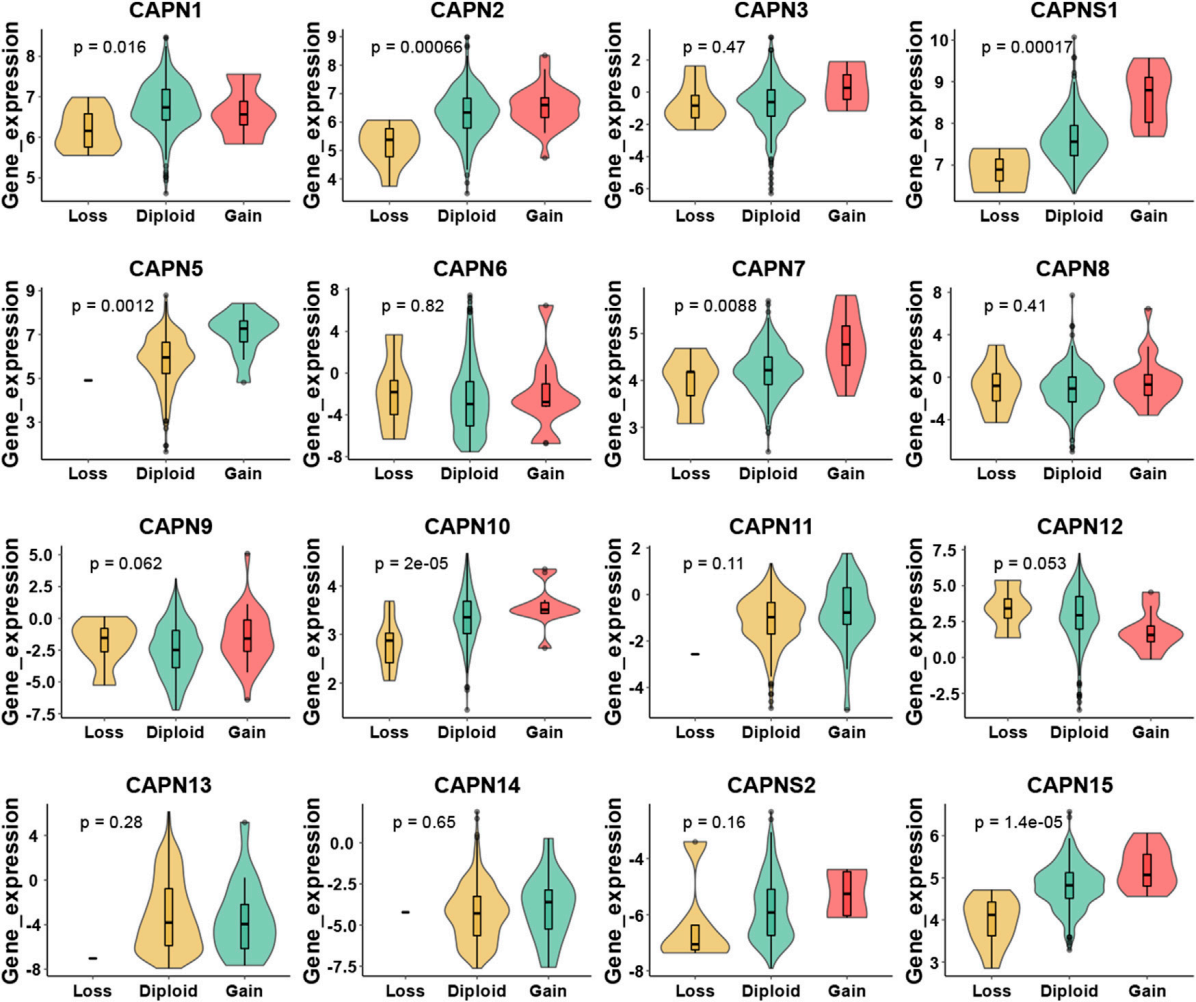
Relationship between CNV and calpain member expression in HCC by violin plots.

**FIGURE 3:**
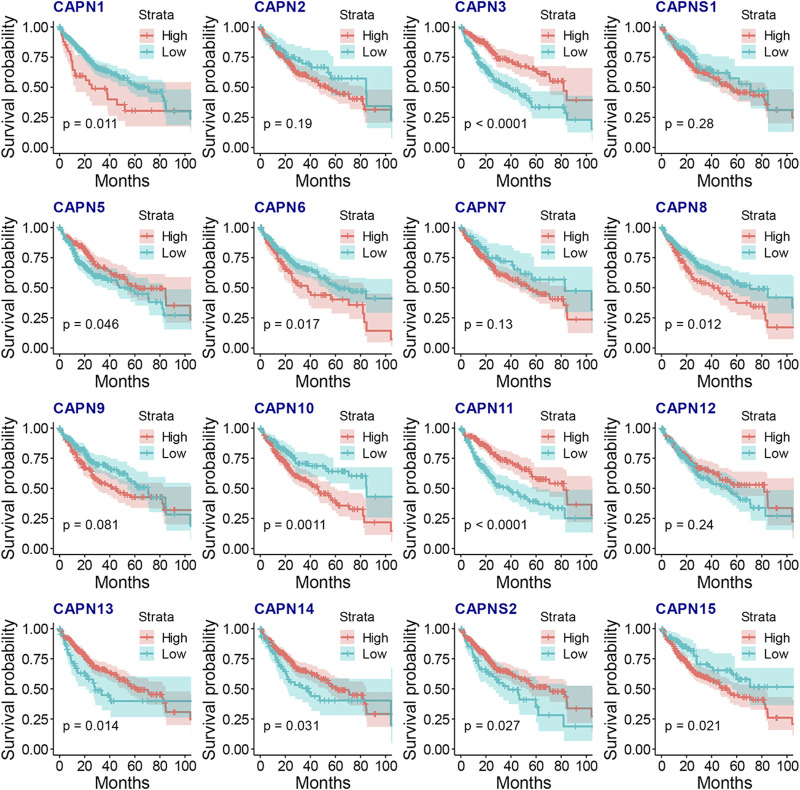
KM plots on association between the gene expression of calpain members and the OS of HCC.

There were 363 HCC samples with read count data collected from the TCGA. We found that CAPN1, CAPN2, CAPN3, CAPNS1, CAPN8, CAPN9, CAPN10, CAPN11, CAPN12, CAPN13, CAPN14, and CAPN15 were upregulated in tumor tissues and CAPN5, CAPN6, CAPN7, and CAPNS2 were downregulated in tumor tissues (Figures 1A, B). The relationship between the expression of calpain members and the HCC clinical factors is listed in Figure 1C. The CNV alterations of calpain members are listed in Figure 1D; Supplementary Table S1. The CAPN9 and CAPN11 showed a higher proportion of gains in CNVs of HCC patients (Figure 1D; Supplementary Table S1, the difference was greater than 5%). We found that nearly half of calpain members had a higher gene expression when the CNV number was high, which comprised CAPN2, CAPNS1, CAPN5, CAPN7, CAPN10, and CAPN15 (Figure 2). We observed that few mutations occurred in calpain members. The mutation rate of each calpain member ranged from 0.28% to 1.42% (Supplementary Table S2). We found the promoter DNA methylation was negatively correlated with gene expression in CAPN1, CAPN5, CAPN6, CAPN12, and CAPN13 (Supplementary Table S3, R square less than −0.3 and *p*-value less than 0.05). In survival analysis of calpain members, we found the higher expression of CAPN7, CAPN11, CAPN5, and CAPNS2 was associated with better OS of HCC and higher expression of CAPN15, CAPN10, CAPN2, CAPN8, and CAPN14 was associated with worse OS of HCC (Figure 3).

### 3.2 The clustering analysis of calpain members in HCC patients

**FIGURE 4:**
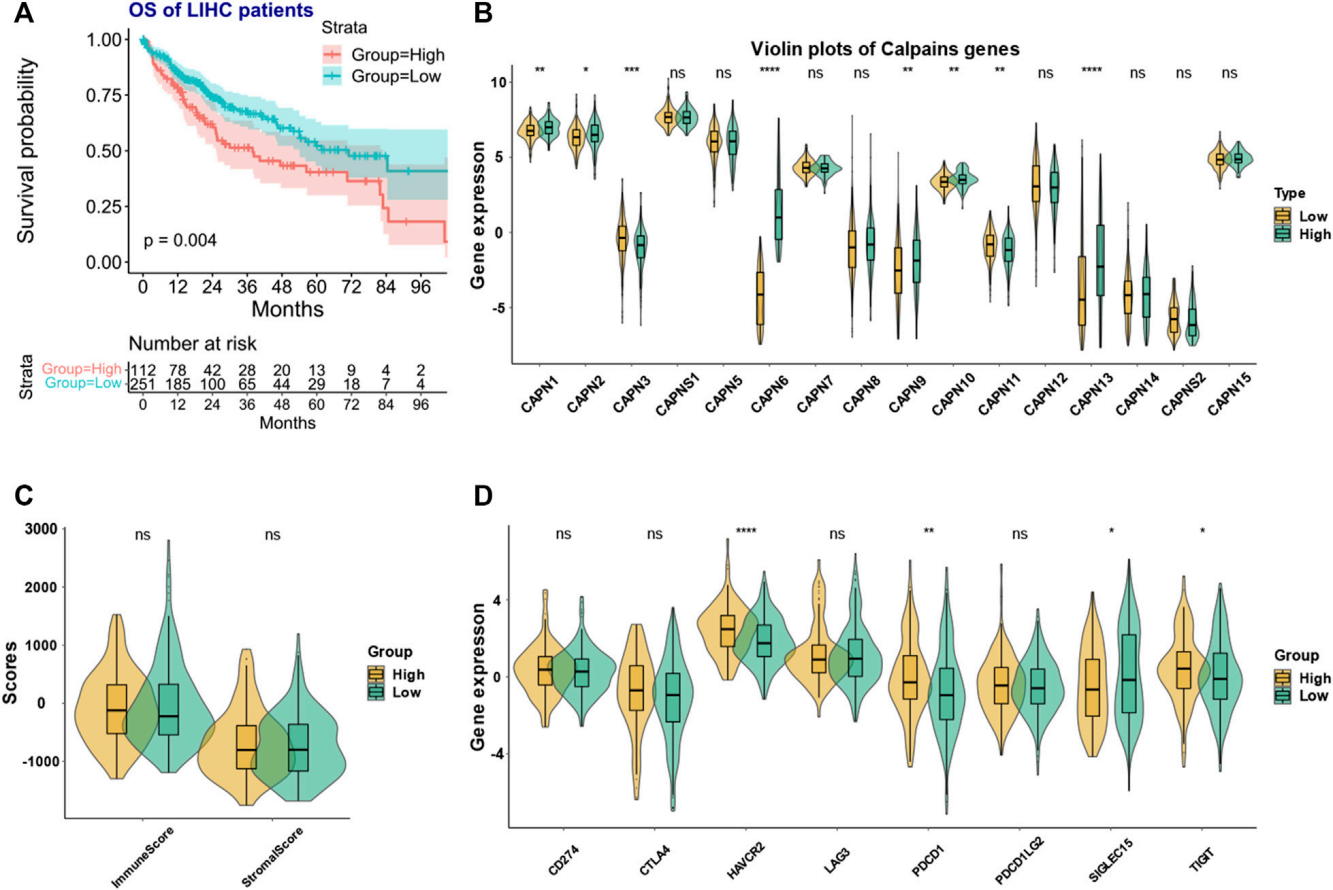
Calpain-related clusters and their relationship with immune and stromal scores and immune checkpoint family genes in HCC **(A)** KM plot of the different calpain-related clusters. **(B)** Expression pattern of each calpain member between the two calpain groups. The asterisks represented the statistical *p*-value (*p < 0.05; **p < 0.01; ***p < 0.001; ****p < 0.0001). **(C)** Violin plots presented the association between calpain groups and immune and stromal scores in TCGA-HCC cohort. **(D)** Expression of eight immune checkpoint family genes between two calpain groups by presented by violin plots.

The clustering analysis identified two groups of HCC patients with similar gene expression pattern while having significant prognostic difference (Figure 4A). We named the two groups as high- and low-risk calpain groups. After analyzing the expression distribution of high- and low-risk calpain groups (Figure 4B), we found that CAPN1, CAPN2, CAPN8, CAPN9, CAPN10, CAPN13, and CAPN15 were both higher expressed in tumor than in normal tissues and higher expressed in the high-risk calpain group than in the low risk calpain group (Figures 1A, B; Figure 4B), and CAPN5, CAPN7, and CAPNS2 were both lower expressed in tumor than in normal tissues and lower expressed in the high-risk calpain group than in the low-risk calpain group (Figures 1A, B; Figure 4B).

### 3.3 The immune microenvironment difference between two calpain-related groups

**FIGURE 5:**
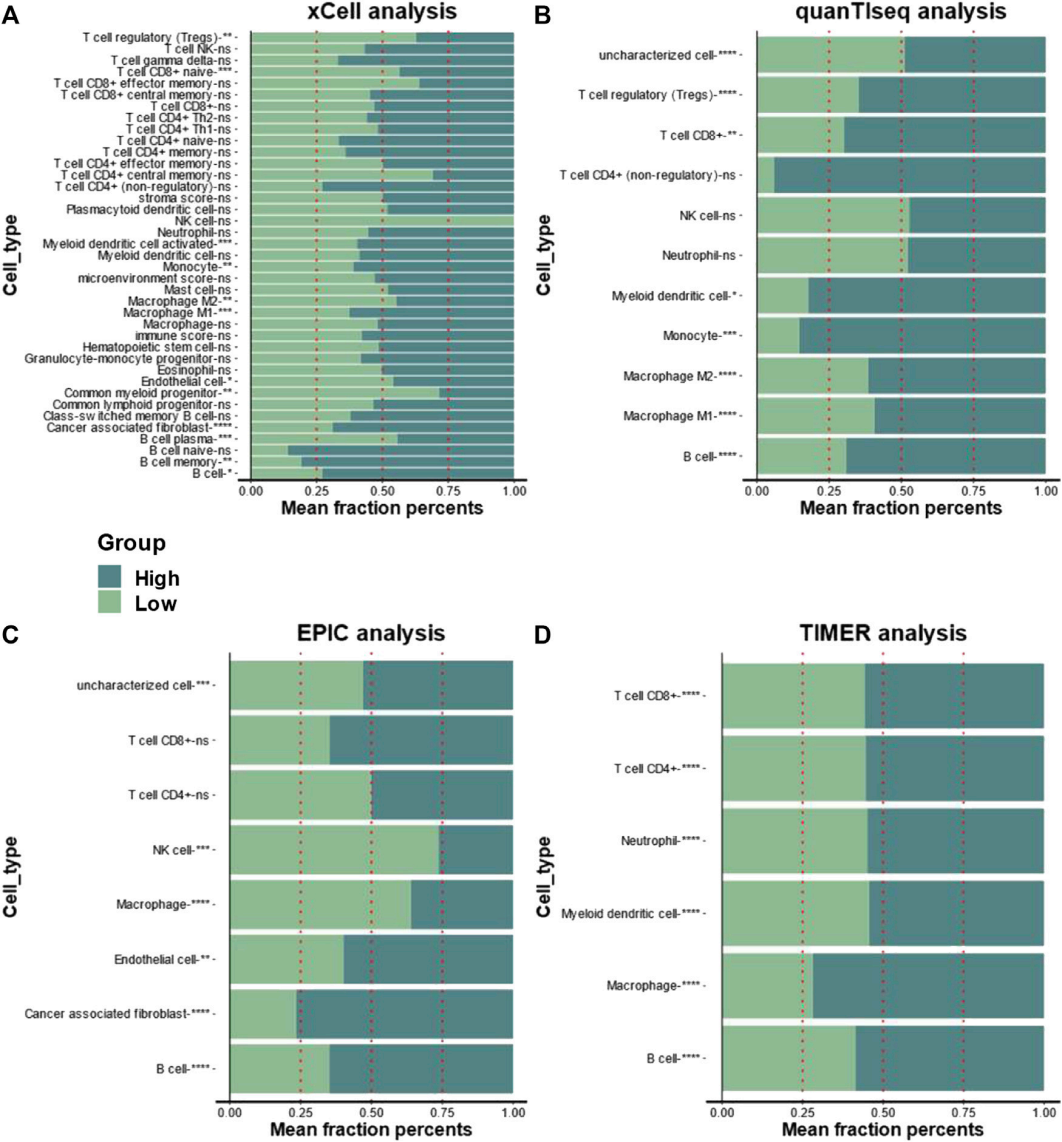
Association between infiltration level of immune cells and the calpain groups in HCC xCell analysis **(A)**, quanTIseq **(B)**, EPIC **(C)**, and TIMER **(D)** analyses of the two calpain groups. The x-axis is the mean cell fraction of specific tumor immune contexture. The asterisks represented the statistical *p*-value (*p < 0.05; **p < 0.01; ***p < 0.001; ****p < 0.0001).

In the ESTIMATE analysis, we found the calpain groups had no association with immune score and stromal score (Figure 4C). In the analysis of eight immune-checkpoint genes, we found that HAVCR2, PDCD1, and TIGHT were significantly higher expressed in the high-risk calpain group while SIGLEC15 was significantly lower expressed in the high-risk calpain group (Figure 4D). In the immune cell fraction analysis, we found that the four different algorithms all showed that B cells were significantly higher in the high-risk calpain group (Figures 5 A–D).

### 3.4 The association between two calpain-related groups and drug response

The CMap analysis found that the histone deacetylases (HDAC) inhibitor trichostatin A and PI3K-AKT-mTOR pathway inhibitors LY-294002 and wortmannin might be the potential drugs targeted to the high-risk calpain group (Table 1, *p*-value < 0.05, FDR < 0.1).

**TABLE 1:** Drugs that had potential therapeutic effects on the high-risk calpain group by CMap analysis.

Drug	*p*-value	FDR
Trichostatin A_MCF7	2.90E-25	1.04E-21
LY-294002_MCF7	9.75E-10	1.75E-06
Wortmannin_MCF7	1.61E-05	0.019222
Trichostatin A_PC3	8.43E-05	0.075585

The significant results of chi-square analysis were bolded. The *p*-value describes the difference between the RT and non-RT group.

### 3.5 The DEG screening between calpain-related groups and subsequent functional analyses

**FIGURE 6:**
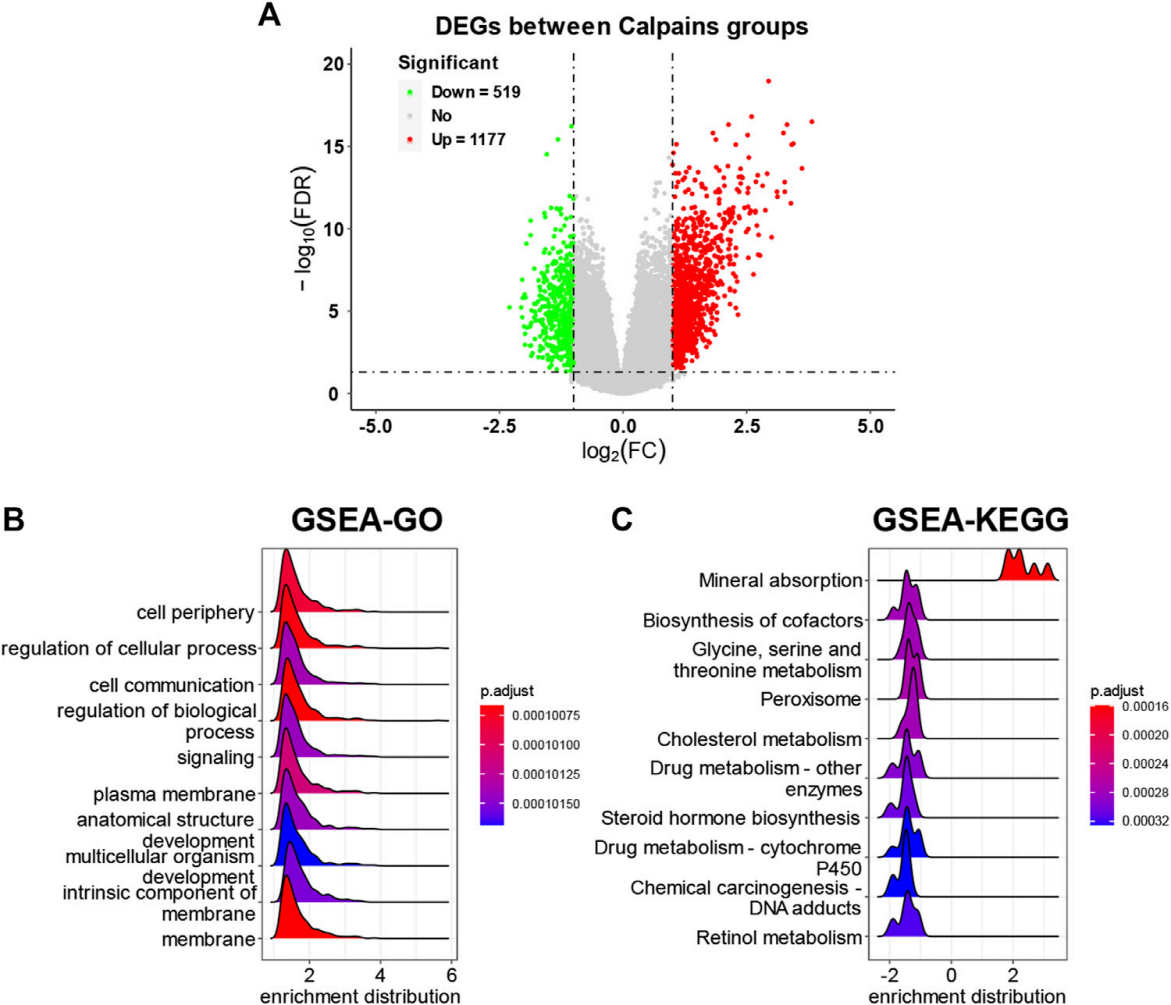
DEG identification and related functional analyses. **(A)** Volcano plot of DEGs between calpain groups. **(B,C)** GSEA of DEGs of GO **(B)** and KEGG **(C)** category.

The DEGs analysis identified 519 downregulated genes and 1177 upregulated genes (Figure 6A; Supplementary Table S4). The GSEA-GO analysis showed that cell periphery, regulation of cellular process, and cell communication were upregulated in the high-risk calpain group (Figure 6B). The GSEA-KEGG analysis found that a series of energy metabolism and biosynthesis pathways were downregulated in the high-risk calpain group, such as glycine, serine, and threonine metabolism, and steroid hormone biosynthesis pathways (Figure 6C).

### 3.6 The prognostic signature selection and prognostic model construction

**FIGURE 7:**
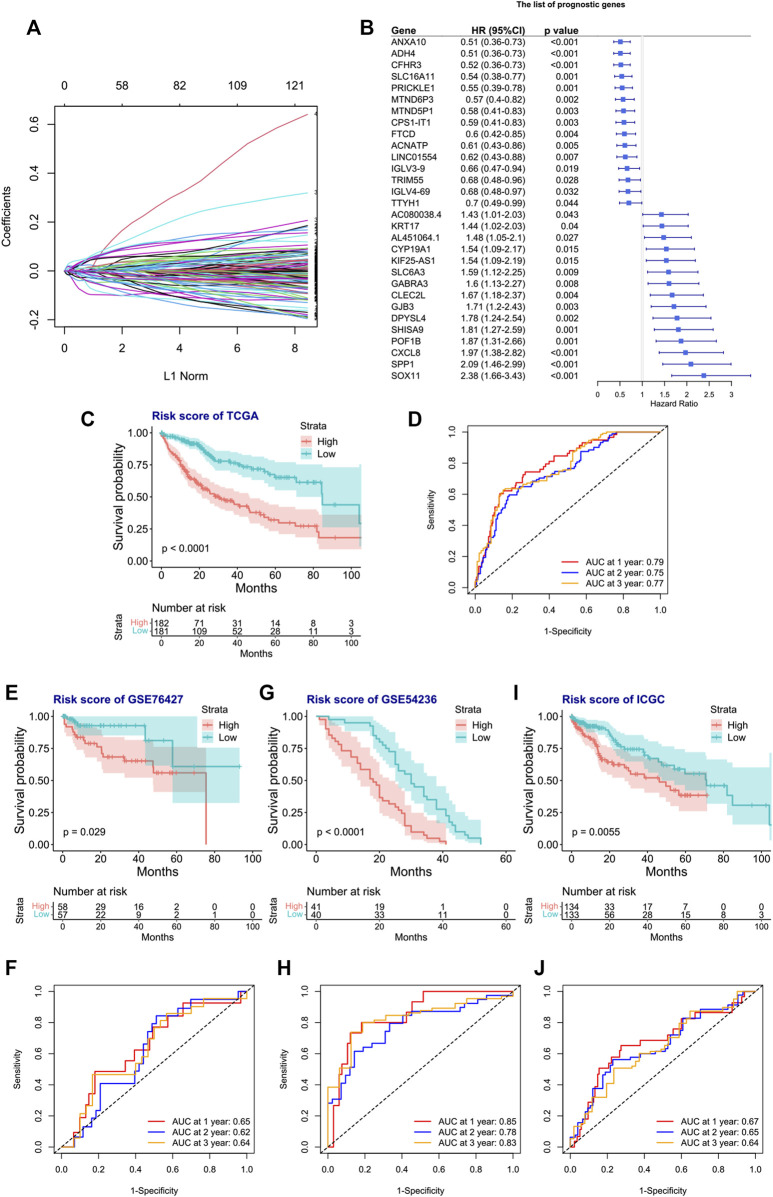
Construction and validation of the risk score model for the OS of HCC patients. **(A)** Selection of the calpain-related hub survival genes by LASSO Cox analysis. **(B)** Forest plot of the hub survival genes in the TCGA database. **(C)** KM plot of the high- or low-risk score HCC patients in TCGA. **(D)** AUC plots of risk score to predict the 1-, 2-, and 3-year OS of HCC in TCGA. **(E–J)** Validations of the KM plot and AUC plot of the risk score model for predicting OS of HCC in the GEO-GSE76427 dataset **(E, F)**, GEO-GSE54236 dataset, **(G, H)**, and ICGC dataset **(I, J)**.

**FIGURE 8:**
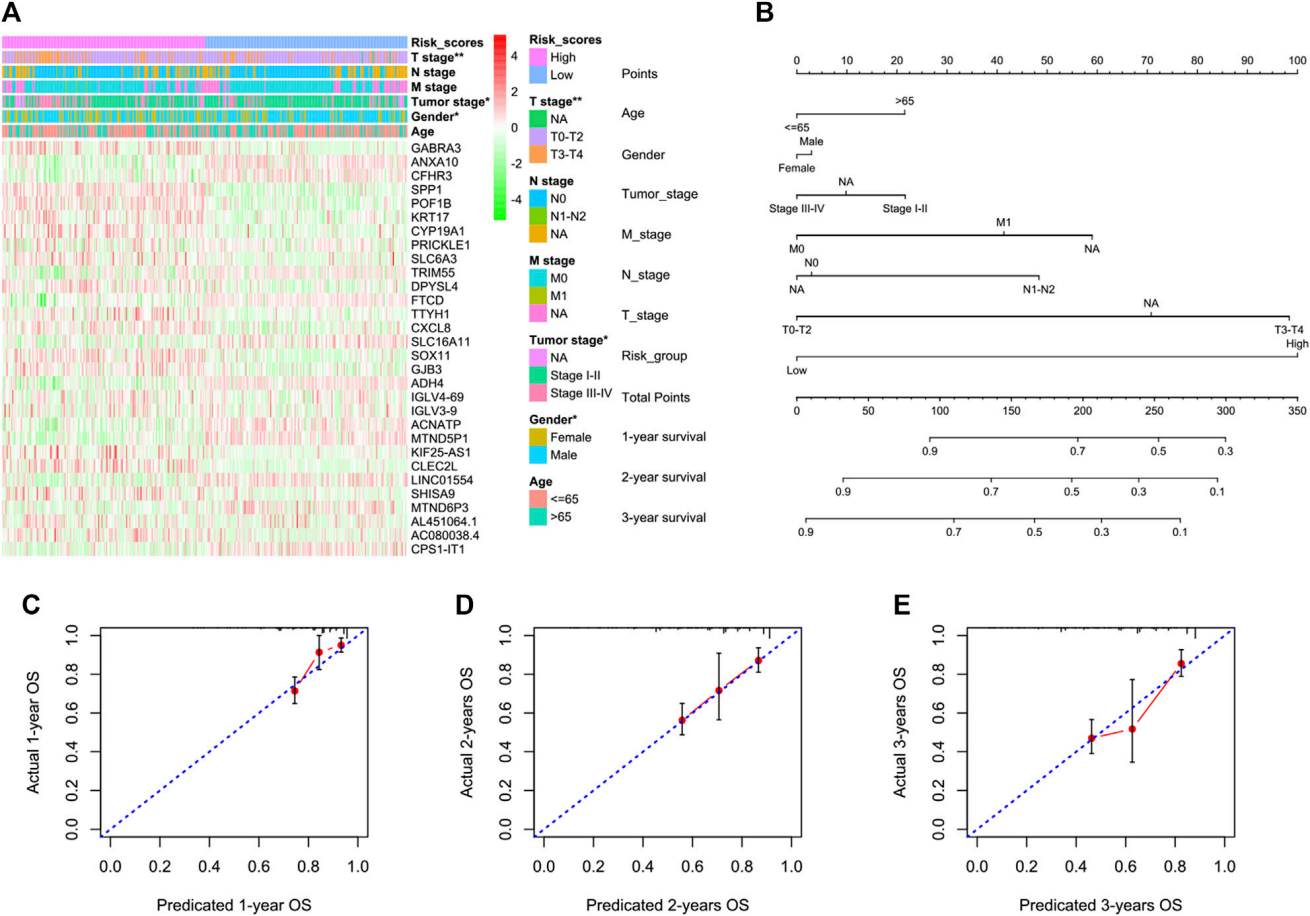
Association between the risk score model and the clinical features of HCC and the construction and validation of a nomogram to predict the 1-year, 2-year, and 3-year OS of HCC in TCGA. **(A)** Heatmap represents the hub survival gene expression pattern in the risk score group and the clinical features of HCC patients. The asterisks represented the statistical *p*-value (*p < 0.05; **p < 0.01). **(B)** Multivariate Cox model-based nomogram of HCC patients in TCGA. **(C–E)** Calibration plots for the internal validation of the nomogram. The x-axis represents the nomogram-predicted OS, and the y-axis represents the actual OS of patients with HCC.

We next tried to discover the calpain-related hub survival genes for HCC. The univariate Cox analysis of each calpain-related DEG identified 217 favorable prognostic genes and 129 inferior prognostic genes for HCC patients (Supplementary Table S5). The prognostic-related genes were then used as an input for LASSO analysis. The LASSO analysis identified 30 hub survival genes (Figure 7A; Supplementary Table S6). The detailed prognostic information of each hub gene was shown in a forest plot (Figure 7B). Based on the hub survival genes, a risk score model was built, which showed that higher risk score had significantly lower OS of HCC patients (Figure 7C). The AUC plot identified that this risk score model had relatively high ability to predict the OS of HCC patients (AUC > 0.7 for 1-year, 2-year and 3-year prediction, Figure 7D). This risk score model was validated in three different external cohorts, GSE76427 (Figures 7E, F), GSE54236 (Figures 7G, H), and ICGC (Figures 7I, J). A heatmap showed the association between hub survival gene expression pattern in the risk score group, and the clinical features of HCC patients, and the risk score was found to be associated with higher T stage and higher tumor stage of HCC patients (Figure 8A). In addition, a nomogram was built to predict 1-, 2-, and 3-year OS of HCC patients, which comprised items of age, gender, TNM stage, and risk score (Figure 8B). The calibration plots showed the nomogram had good prediction ability to the OS of HCC patients (Figures 8C–E).

## 4 Discussion

The calpain family members have numerous substrates, some of which were well-known to promote cancer progression, such as the tumor suppressor P53; signaling molecules PKC and FAK; proto-oncogenes c-fos and c-jun; and apoptosis proteins caspase-3, -7, -8, -9, and -12 (Nian and Ma, 2021). The calpain system was also identified to be associated with HCC progression. The hepatitis C virus (HCV) protein was found to activate calpains to degrade the BH3 interacting domain death agonist (Bid) and thus inhibit the apoptotic signaling. The inhibition of calpains would enhance the immune-mediated elimination of HCV-infected cells (Simonin et al., 2009) and also attenuate MMP-2 and MMP-9 secretion and decrease the metastatic ability of HCC cells (Chen et al., 2013). The overexpression of CAPN2 and CAPNS1 was found to promote HCC progression by the activation of oncogene signaling pathways (Dai et al., 2014; Ma et al., 2022). However, in the field of multi-omics, there was no comprehensive analysis of calpain members and HCC progression and prognosis. Our study found that the expression of 14 out of 16 calpain members was significantly different between tumor and normal tissues in HCC patients. The clustering analysis identified two calpain clusters with survival difference. The prognostic analysis revealed calpain-related signature, which had good prediction for the OS of HCC.

Recently, immunotherapy, such as ICIs, has achieved various successes in the treatment of cancers, including HCC (Solimando et al., 2022; Viscardi et al., 2022). However, the improvement of OS was limited. The tumor immune microenvironment was found to influence the tumor immune escape and immunotherapeutics efficacy of HCC (Khanam and Kottilil, 2022). Hence, exploring the HCC immune environment and its interaction with ICIs is important. In our study, we found the calpain groups were associated with the immune microenvironment. We found that the immune checkpoint genes PDCD1, HAVCR2, and TIGIT had a high expression level in the high-risk calpain group, which indicated that the high-risk calpain group might benefit from ICI therapy by blocking specific immune checkpoints. The immune cell fraction analysis found that the B cells were higher in the high-risk calpain group. A systematic review found that B cells played a beneficial role in most types of cancers (Wouters and Nelson, 2018). The HCC patients who had both CD3^+^ and CD20^+^ tumor-infiltrating cells were observed to have a more favorable prognosis than those who had tumor-infiltrating cells with either CD3^+^ or CD20^+^ (Garnelo et al., 2017). Another study found the tumor-infiltrating CD20^+^ B cells were predominantly concentrated in the tumor invasive margin. It is observed that high density of margin-infiltrating B lymphocytes positively correlated with small tumor size, absence of vascular invasion, increased density of CD8^+^ T cells, and better OS of HCC patients (Shi et al., 2013). On the other hand, a previous study found that regulatory B cells accelerated HCC progression via the CD40/CD154 signaling pathway (Shao et al., 2014). Our study proved new insight into the association between B cells and HCC, the underlying mechanism of calpains and the B cells, and HCC requires further studies to explore.

The histone deacetylases (HDACs) play an essential role in the regulation of transcription (Kulka et al., 2020). HDAC activation was widely identified to be associated with the resistance of chemotherapy, targeted therapy, and immune therapy in cancers (Mamdani and Jalal, 2020). In HCC, HDAC members were found to promote cell proliferation migration and invasion through different signaling pathways such as the TGF-β1, PTEN, and estrogen receptor α signaling pathways (Xue et al., 2020; Guo et al., 2021; Wei et al., 2021). A series of HDAC inhibitors were identified to inhibit HCC growth and metastasis, such as panobinostat and SAHA analogs (Xia et al., 2022). Furthermore, the HDAC inhibitor was observed to lower the CAPN1 mRNA expression (Mochalova et al., 2020), and downregulation of HDAC2 transcriptionally decreased CAPN2 expression by hyperacetylation of the promoter of the CAPN2 gene (Siuda et al., 2019). The CMap analysis of our study found that HDAC inhibitors trichostatin A and vorinostat had a potential therapeutic effect on HCC patients in the high-risk calpain group, which had higher CAPN1 and CAPN2 expression. The PI3K/Akt/mTOR pathway was found to play an important role in the progression of HCC in processes like proliferation, metastasis, chemotherapy and radiotherapy resistance, and energy metabolism (Liu et al., 2016). A previous study found that CAPN6 was downregulated by the PI3K inhibitor or Akt deletion (Liu et al., 2011), and miR-449a could promote HCC cell apoptosis by downregulation of CAPN6 (Liu et al., 2016). Our CMap analysis showed that the PI3K inhibitors LY-294002 and wortmannin might be potential drugs targeted to high-risk calpain group HCC patients, with higher CAPN6 expression. Our drug sensitivity analysis provided new insight into the association between calpains and the PI3K-AKT pathway and HDACs in HCC, which require further study to confirm.

We identified the DEGs between calpain groups and subsequently performed the functional enrichment analysis. We found a series of metabolism pathways were downregulated in the high-risk calpain groups, such as cysteine, methionine, cholesterol, glycine, serine, and threonine. A previous study found that energy metabolism-related pathways and biological process were upregulated in the group with longer survival in bladder cancer (Zhang et al., 2022), which was consistent with our results.

We further identified 30 calpain-related hub survival genes in HCC. The risk model based on the hub survival genes was significantly associated with HCC prognosis in four different datasets. Our nomogram that included the risk model also showed good prediction ability to the OS of HCC. There were 15 new identified genes that we found, and no previous study had shown an association between these genes and HCC, i.e., SLC16A11, MTND6P3, MTND5P1, ACNATP, LGLV3-9, LGLV4-69, TTYH1, AC080038.4, AL451064.1, KIF25-AS1, CLEC2L, GJB3, DPYSL4, SHISA9, and POF1B. For the rest of hub survival genes, there were 13 out of 15 genes which had consistent results with the findings of a previous study, of which for these survival favorable hub survival genes, immunohistochemistry assay identified that higher ADH4 protein expression was associated with favorable OS of HCC (Wei et al., 2012); Overexpression of PRICKLE-1 (Chan et al., 2006), FTCD (Chen et al., 2019), and LINC01554 (Hao et al., 2020) suppressed HCC cell proliferation; overexpression of TRIM55 inhibited migration and invasion of HCC cells (Li et al., 2019); knockdown of CFHR3 promoted proliferation, migration, and invasion of HCC cells (Wan et al., 2022); melatonin suppressed HCC progression via upregulation of lncRNA-CPS1-IT-mediated HIF-1α inactivation (Wang et al., 2017). For these unfavorable hub survival genes, previous studies found that overexpression of CXCL8 in HCC cell resulted in increased cell proliferation and migration (Yang et al., 2020); the impaired expression of GABRA3 (Liu et al., 2008) and SPP1 (Wang et al., 2019) decreased HCC cell viability; ITGBL1 increased KRT17 overexpression and promoted the metastatic ability of HCC cells (Huang et al., 2020); nicotine stimulated CYP1A1 expression to promote HCC cell proliferation (Ung et al., 2021); carvacrol inhibited the viability of HCC cells by downregulating SLC6A3 (Yin et al., 2022). In summary, our study provided new insights between these genes and calpains and the prognosis of HCC.

Our study had several limitations. First, despite the hub survival gene signature had well prediction ability in four different datasets, the data from TCGA, ICGA, and GEO were all retrospective designed, and with much incomplete information; therefore, it is possible that the quality and accuracy of the data may vary. Second, the sample size of the study was limited, and further studies with larger sample sizes are needed to validate our findings and to provide a more comprehensive understanding of the associations between calpain family members and HCC progression and prognosis. Third, our study only focused on the classical and non-classical calpain members and CAPNS1–2. Future studies may seek to explore the role of other calpain family members in HCC and to determine whether they are associated with HCC prognosis and therapy response. Fourth, the underlying mechanisms between calpains and hub survival genes and HCC progression needs further *in vitro* or *in vivo* studies to determine whether these genes can be targeted for therapeutic purposes in HCC patients.

## 5 Conclusion

This is the first comprehensive bioinformatics study of the calpain members in HCC. The clustering analysis showed groups with different expression patterns of calpain members had significantly different OS in HCC. We found PI3K-AKT and HDAC inhibitors had a potential treatment effect on the high-risk calpain group. Furthermore, a calpain-related signature which had good prediction ability for HCC OS was constructed and validated in different external datasets. Future studies are required to confirm our findings between calpains and HCC progression and prognosis.

## Data Availability

The original contributions presented in the study are included in the article/Supplementary Material, further inquiries can be directed to the corresponding authors.

## Supplementary material

SUPPLEMENTARY TABLE S1 Incidence rate of CNVs of calpain members in the TCGA-HCC cohort.

SUPPLEMENTARY TABLE S2 Incidence rate of mutations of calpain members in the TCGA-HCC cohort.

SUPPLEMENTARY TABLE S3 Correlation analysis between DNA methylation and RNA expression in each calpain member in TCGA-HCC.

SUPPLEMENTARY TABLE S4 DEGs identified between calpain groups in the TCGA-HCC cohort.

SUPPLEMENTARY TABLE S5 Survival-related DEGs in the TCGA-HCC cohort.

SUPPLEMENTARY TABLE S6 Coefficients of each survival-related DEG by LASSO analysis.
